# Velocity of Nervous Force

**Published:** 1863-12

**Authors:** 


VELOCITY OF NERVOUS FORCE.—The "Veterinarian" states that by the aid of a chronoscope M. Hirsch has come to the conclusion that nerves transmit their impressions at the rate of thirty-four metres a second. M. Heinholtz estimates the velocity at 190 feet per second, but his experiments were performed on the motor nerves of a frog, and those of M. Hirsch on the sensitive nerves of a man.

